# Supplementary material for: Incidence, persistence, and clearance of cervical human papillomavirus infection among gynecological outpatients in Kunming, Yunnan, China, 2019–2023: a retrospective cohort study
Source: PeerJ. 2025 Nov 4;13:e20215. doi: 10.7717/peerj.20215 (PMC12593720; doi:10.7717/peerj.20215)
Supplement: Supplemental Information 3 [file peerj-13-20215-s003.docx]

**Patient ID:** The unique identifier assigned to the patient’s medical visit card at the time of hospital registration.

**Barcode:** The unique identifier assigned to the patient’s sample during laboratory testing. A single Barcode is associated with the detection of 25 HPV genotypes.
