# Supplementary material for: Incidence, persistence, and clearance of cervical human papillomavirus infection among gynecological outpatients in Kunming, Yunnan, China, 2019–2023: a retrospective cohort study
Source: PeerJ. 2025 Nov 4;13:e20215. doi: 10.7717/peerj.20215 (PMC12593720; doi:10.7717/peerj.20215)
Supplement: Supplemental Information 4 [file peerj-13-20215-s004.docx]

Supplementary Table 1. Follow-up outcomes for baseline HPV-positive patients (n, %) and proportion involving high-risk HPV genotypes (n, %)

| Follow-up outcome among baseline HPV-positive patients | Total n (%) | High-risk HPV involved n (%) |
| --- | --- | --- |
| Co-infection with additional genotypes  (original genotypes still detected) | 136(58.37%) | 106(77.94%) |
| Complete genotype replacement  (original genotypes cleared, new genotypes detected) | 97(41.63%) | 85(87.63%) |

Figure S1. Newly detected HPV genotypes at second screening. The stacked bars show the number of cases for each genotype, divided into co-infection (new genotypes added while baseline types were retained) and complete genotype replacement (baseline types cleared, only new genotypes detected).
