# Supplementary figures and images for: Incidence, persistence, and clearance of cervical human papillomavirus infection among gynecological outpatients in Kunming, Yunnan, China, 2019–2023: a retrospective cohort study
Source: PeerJ. 2025 Nov 4;13:e20215. doi: 10.7717/peerj.20215 (PMC12593720; doi:10.7717/peerj.20215)

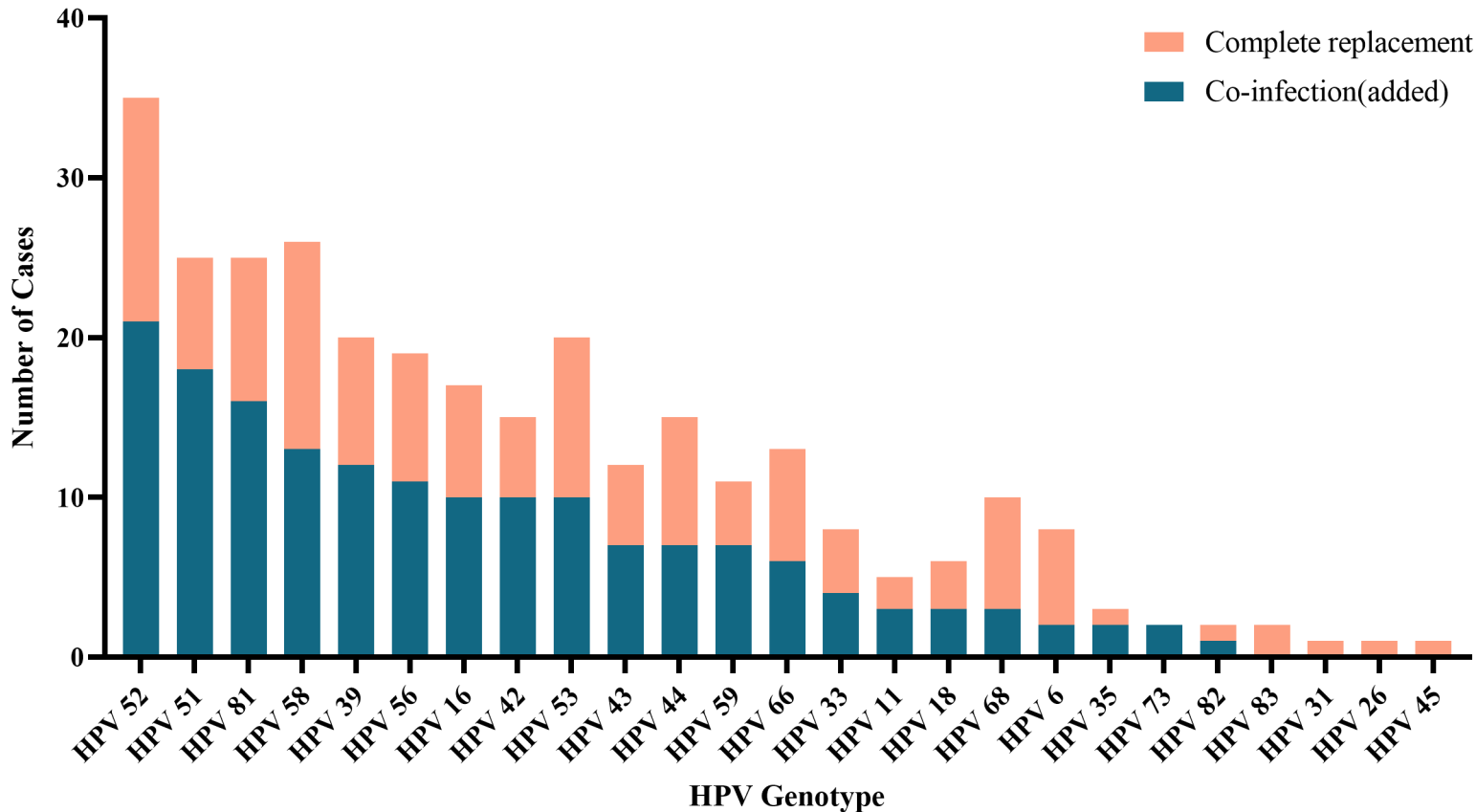

Supplement: Supplemental Information 5 — The stacked bars show the number of cases for each genotype, divided into co-infection (new genotypes added while baseline types were retained) and complete genotype replacement (baseline types cleared, only new genotypes detected). [file peerj-13-20215-s005.pdf]
